# An agenda for future research regarding the mental health of young people with care experience

**DOI:** 10.1111/cfs.13015

**Published:** 2023-03-05

**Authors:** John Devaney, Luke Power, Paula Jacobs, Gavin Davidson, Rachel Hiller, Joanna Martin, Claire McCartan, Pearse McCusker, Rosie McGuire, Alice Phillips, Autumn Roesch‐Marsh, Anita Thapar

**Affiliations:** ^1^ School of Social and Political Science University of Edinburgh Edinburgh UK; ^2^ Health Sciences University of Stirling Stirling UK; ^3^ School of Social Sciences, Education and Social Work Queen's University Belfast Belfast UK; ^4^ Department of Psychology University of Bath UK; ^5^ Clinical, Educational & Health Psychology University College London UK; ^6^ Wolfson Centre for Young People's Mental Health, Division of Psychological Medicine and Clinical Neurosciences Cardiff University UK

**Keywords:** care experience, children, mental health, neurodiversity, out‐of‐home care, young people

## Abstract

Young people who are currently or were previously in state care have consistently been found to have much higher rates of mental health and neurodevelopmental difficulties than the general youth population. While a number of high‐quality reviews highlight what research has been undertaken in relation to the mental health of young people with care experience and the gaps in our knowledge and understanding, there is, until now, no consensus, so far as we aware, as to where our collective research efforts should be directed with this important group. Through a series of UK wide workshops, we undertook a consultative process to identify an agreed research agenda between those with lived experience of being in care (*n* = 15), practitioners, policy makers and researchers (*n* = 59), for future research regarding the mental health of young people with care experience, including those who are neurodiverse/have a neurodevelopmental difficulty. This consensus statement identified 21 foci within four broad categories: how we conceptualize mental health; under‐studied populations; under‐studied topics; and underused methodologies. We hope that those who commission, fund and undertake research will engage in this discussion about the future agenda for research regarding the mental health of young people with care experience.

## 1 INTRODUCTION

Of all the things we could do, what is most needed? Internationally, it is estimated that between 0.5% and 1.0% of children live in institutional formal out‐of‐home care, with a further group of children cared for within family settings by both kinship and nonkinship carers (Desmond et al., 2020). In the United Kingdom (UK), approximately 90 000 children are currently in state care (McGhee et al., 2017), with a larger proportion of the child population experiencing some form of state care at some point in their life. Children come into state care for a variety of reasons and through a number of different legal routes. However, there is a common thread of children having experienced a range of adversities, including maltreatment, loss, parental mental ill‐health and poverty, which shape their pre‐ and in‐care experiences, and impact on children's mental health and well‐being (Baldwin et al., 2019). In the United Kingdom, most children enter care from school age or older, meaning in many instances, these adverse experiences have been particularly prolonged. Indeed, adolescents are the fastest growing age group currently entering the UK care system (Clarke & Penington, 2021).

As noted by Jacobs et al. (n.d.), although there are increasing concerns about the mental health and well‐being of children in general (Frith, 2016), there are concerns about children with care experience in particular. Children who are currently or were previously in state care have consistently been found to have much higher rates of mental health and neurodevelopmental difficulties such as attention‐deficit hyperactivity disorder (ADHD) or autism spectrum disorder than the general youth population (Seker et al., 2021). There is good international evidence of the types of mental health outcomes experienced by this group. Yet, there is a proportional dearth of evidence on how to address their needs. This is in part due to the complex array of factors impacting on children's mental health outcomes, such as a neurodevelopmental condition and the lack of a more comprehensive and cohesive approach to addressing the gaps in our understanding about a range of associated issues. This complexity and the importance of supporting young people's positive mental health has been a feature of the independent reviews of children's social care in England (McAllister, 2022) and Scotland (Duncan, 2020).

This article presents the findings of a consultative process to seek to identify an agreed research agenda between those with lived experience of being in care, practitioners, policy makers and researchers, for future research regarding the mental health of young people with care experience, including those who are neurodiverse (Sonuga‐Barke & Thapar, 2021). Although there are a number of high‐quality reviews highlighting what research has been undertaken in relation to the mental health of young people with care experience and the gaps in our knowledge (e.g., Luke et al., 2014), there is, until now, no consensus, so far as we aware, as to where our collective research efforts should be directed with this important group. The work reported in this article was underpinned by direct work with young people with care experience and seeks to highlight future directions for research to support policy and practice. While the work has a specific focus on the United Kingdom, there are implications for other jurisdictions.

### 1.1 The mental health of young people with care experience

Childhood and adolescence are crucial periods for developing social and emotional habits important for mental well‐being. These include adopting healthy sleep patterns; exercising regularly; developing coping, problem solving and interpersonal skills; and learning to manage emotions. While there is robust evidence to support the role of genetics in specific mental disorders, protective and supportive environments in the family, at school and in the wider community are also important for developing and maintaining positive mental health and in helping young people to adapt to and be resilient against some types of adversities experienced (de Pablo et al., 2020). The World Health Organization (2022) has defined positive mental health as “… a state of mental well‐being that enables people to cope with the stresses of life, realize their abilities, learn well and work well, and contribute to their community. It is an integral component of health and well‐being that underpins our individual and collective abilities to make decisions, build relationships and shape the world we live in. Mental health is a basic human right. And it is crucial to personal, community and socio‐economic development.”

As noted by Luke et al. (2014), it is important to remember that the increased risk of neurodevelopmental (e.g., ADHD, autism) and mental health problems for children and adults with care experience cannot be solely attributed to their experiences of maltreatment and neglect within their families of origin. However, there is robust evidence that whether judged categorically or dimensionally, children looked after by local authorities in the United Kingdom have significantly poorer mental health than the most disadvantaged children outside the care system (Ford et al., 2007). In addition, neurodevelopmental disorders, such as autism spectrum disorder, ADHD, and learning and communication disorders are elevated in care‐experienced populations (Ford et al., 2007), and although they should not be considered as mental health problems, this group are an especially high‐risk group for mental health problems such as anxiety and depression.

The factors contributing to the presentation of strengths and difficulties for any individual with care experience are likely to be complex and specific to that person. However, there is now significant international consensus of the heightened risk of experiencing immediate and ongoing mental health difficulties for children who have experience of state care. For example, Seker et al. (2021) undertook a systematic review and meta‐analysis of studies to estimate the prevalence rates for mental health disorders among adults with a foster or residential child welfare or juvenile justice care history, comparing them where possible with rates among the general population. Most of the included 19 studies were prospective and examined mental health disorders among adults with a child welfare out‐of‐home care placement history. Adults with a foster or residential care history resulting from child welfare placement showed significantly higher rates of any mental health disorder (odds ratio [OR] = 1.56, 95% CI [1.14, 2.13], *p* = 0.0061) compared with the general population. The meta‐analysis revealed a pooled prevalence rate of 30% for any mental health disorder in adults with a foster or residential child welfare placement history. For specific disorders, prevalence rates for depressive disorders (OR = 1.98, 95% CI [1.28, 2.89], *p* = 0.002), substance use disorders (OR = 1.33, 95% CI [1.16, 1.91], *p* < 0.001), and anxiety disorders (OR = 1.75; 95% CI [1.20, 2.56], *p* = 0.004) were higher among adults with a foster or residential care history in a child welfare context than in the general population.

As Ford et al. (2007) note, children in the care of local authorities have a higher prevalence of educational and neurodevelopmental difficulties than those disadvantaged and nondisadvantaged children living in private households. In relation to children currently in the care system, Hiller, Fraser, et al. (2023) found that, based on carer reports over the first 3 years of entry to the UK care system, while many young people were relatively resilient to their early experiences and showed low levels of problems, approximately half of the sample were on more problematic, often chronic, trajectories as measured by the Strength and Difficulties Questionnaire. Where young people were experiencing problems in their first year in care, recovery profiles were less common, and chronic problems were relatively robustly associated with instability in care, thereby incurring both personal and economic consequences. Number of placements and being separated from siblings were associated with greater difficulties. As the authors conclude, entry into the care system, where physical safety is the key aim, is not enough of an intervention to expect natural recovery from mental health difficulties or to begin the process of recovery without additional support.

Although we have relatively good evidence of the poor mental health and broader well‐being outcomes of these young people, there remains limited high‐quality empirical evidence on key areas required to effectively address the difficulties experienced by this group. Recent reviews of the extant research (e.g., Luke et al., 2014; NICE, 2021) have highlighted challenges in ensuring that the needs of young people with care experience are understood and met.

Luke et al. (2014) have helpfully differentiated between the effects of ‘add‐on’ interventions (e.g., therapeutic services or mentoring), and the effects of variations in the quality of ‘ordinary care’ provided (e.g., whether the foster placement is a good one). Differences within ordinary care can be a powerful influence on well‐being for children in residential and foster care, as well as providing the context for any additional interventions. Overall, their review of the general literature on care suggests the importance of positive aspects of ordinary care in predisposing children in state care to benefit from interventions targeted at improving mental health and well‐being should not be underestimated.

The evidence to date highlights the importance that high‐quality caregiving, with added evidence informed interventions targeted either directly at the child or indirectly (through the carer or those around the child), providing support where necessary, has the potential to effect positive change in children's well‐being. Additionally, these young people share more commonalities than differences with their peers who are not in care, and it is important to recognize that in spite of some specific adverse experiences, many of the mental health and well‐being interventions that are seen as effective with the general child population are also likely to be successful with this group (e.g., Hiller, Lehmann, et al., 2023). Similarly, there is a growing evidence of the efficacy of particular universal and selective interventions in promoting young people's positive mental health (de Pablo et al., 2020). However, similar to the NICE review (2021), our understanding about how to prevent and address poor mental health for young people with care experience in the immediate and longer term is incomplete.

In summary, there is evidence that some children in care do well despite the adversity they have experienced and the challenging circumstances they have had to contend with. This is often assumed to reflect their ‘resilience’, though this term is hard to define consistently, and may be masking other factors that are likely to be supportive of the young person. As Luke et al. (2014) note, more attention could be given to what promotes positive outcomes, rather than the current over‐emphasis on challenging or problematic behaviour.

Finally, there is a now a substantial body of research indicating that children and young people in care would not want research on outcomes to be restricted to mental health, but also want studies about them doing well on their own terms (Bakketeig et al., 2020).

## 2 RESEARCH AIMS

Over the past decade, there have been a number of high‐quality reviews looking at the existing evidence‐base relating to the mental health of individuals with care experience (e.g., Luke et al., 2014; Seker et al., 2021). These have identified and summarized what we already know and the gaps in our knowledge. However, there is a lack of consensus among researchers, practitioners and policy makers about which of these research gaps could most usefully be addressed in the next 10 years. Additionally, those with care experience have often felt excluded from discussions about what research is necessary. This raises the question of what is it that we need to know, and what could make a difference for young people?

This paper reports on the findings of part of a larger project looking at the mental health needs of adolescents with care experience. This element of the project was designed to facilitate a discussion among a broad coalition of individuals with care experience, practitioners, policy makers and researchers on priorities for future research regarding the mental health of young people with care experience.

## 3 METHODS

The main data collection method used for this exploratory descriptive study were four workshops for researchers, practitioners working in services with care‐experienced young people and policy makers. The four workshops were informed by work with a group of young people with care experience. Ethical approval for the study was provided by the School of Social and Political Science at the University of Edinburgh.

As noted by Smales et al. (2020), the voices of young people with care experience have largely been absent or under‐represented in research examining their health. Studies have rarely involved directly engaging with young people about their perspectives on what health means to them (Bakketeig et al., 2020). Where young people have been involved, studies have typically relied on carers' reports or self‐report questionnaires that primarily examined prevalence of specific mental health or other health conditions. We worked with two organizations involved in supporting young people with care experience—Who Cares? Scotland, and the Camphill School Aberdeen—to bring together 15 young people currently or formerly in care, to help us think about how we should conceptualize and define mental health. These young people also reviewed the draft findings from our work and offered feedback on refinements. The detail of this work will be presented in a subsequent article, but the key messages are presented below.

In our funding application, we had originally planned to convene four in‐person workshops, one in each part of the United Kingdom, to bring practitioners, policy makers and academics together to discuss similar core questions. The UK consists of four separate legal jurisdictions with differing arrangements for the care and support of young people. While the care systems in each UK nation (England, Northern Ireland, Scotland and Wales) have many commonalities, it is also important to recognize that the needs of children, their families and services supporting them may differ due to local context and the legislative arrangements in each country.

However, due to the Covid‐19 pandemic, the fieldwork needed to take place online. As such, we held four online workshops in June and July 2021 with participants invited based on their expertise in relation to young people with care experience and/or young people's mental health. This included researchers, policy makers and those who worked in services for young people with care experience and/or mental health difficulties. Participants were purposively selected from across the four UK countries in recognition of the different legal and organizational arrangements between each country. Through individual contacts, we identified key individuals responsible for policy in relation to state care and/or young people's mental health within each government. We also identified, by looking at respondents to government consultations on the care system in each country, and through our own contacts from previous research, key organizations providing services to or who advocated on behalf of children in state care. Finally, we identified UK‐based researchers who had published empirical studies about children or adults with care experience, or adolescent mental health in the last 5 years in key journals.

We wrote to key individuals (*N* = 111) to invite then to partake in the research due to their role and expertise and also asked them to nominate others who they felt would have views on the future research agenda for this population.

Adapting to the flexibilities of online workshops for participation, each of the four events had a specific focus related to our preliminary work with young people with care experience, and our initial review of existing reviews undertaken in the United Kingdom of the research evidence on the mental health of young people with care experience (Frith, 2016; Luke et al., 2014; Moriarty et al., 2016; NICE, 2015), as follows:

*Outcomes*—what are the mental health and well‐being outcomes we are seeking for care‐experienced young people while in care and after they leave the care system?
*Pathways*—what are the factors impacting on both poor and positive mental health as young people enter, move through and leave the care system?
*Neurodiversity*—how does the care system meet the needs of young people who are neurodiverse (e.g., autism spectrum disorder and ADHD), alongside addressing any adversity and trauma they may have experienced?
*Structural and systemic issues*—how do responses to supporting the mental health of young people with care experience incorporate the likely impact of structural issues in society, such as poverty and racism, and ensure that the child welfare and mental health systems provide child centred support?

**FIGURE 1:**
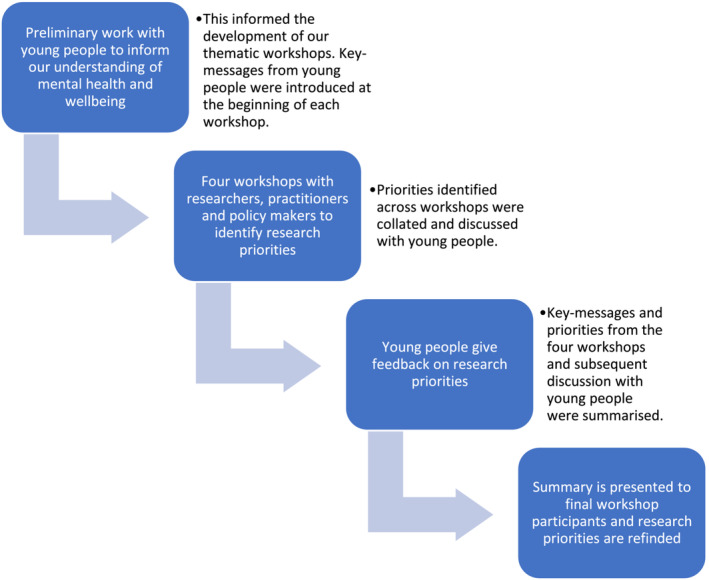
Stages in involvement of young people with care experience in identifying priorities.

We invited potential participants to join a specific workshop given their area of expertise, while also providing them with the option of joining more than one should they wish. The workshops started with an overview of the project, the feedback provided by young people and a short presentation by one member of the research team on the specific focus of the workshop. Participants were then assigned to breakout rooms, with a member of the research team who took detailed notes of the ensuing discussion about existing research related to the topic and potential future research needs. After a short break, participants joined a plenary session with feedback from each group and a wider discussion of the most pertinent issues. The data from the workshops consisted of detailed notes from each of the breakout rooms and the plenary. These were analysed separately by the first and second authors drawing upon the six‐stage, iterative and reflective approach to thematic analysis developed by Braun and Clarke (2006): familiarization; initial coding; identifying themes; reviewing themes; defining themes; evidencing themes in the final write‐up. Initial themes identified individually were then compared, before the initial analysis was discussed with the research team and refined further. Key messages from the four workshops were brought back to the young people to hear their views, sense check initial findings and provide further feedback and refinement (Figure 1).

We then convened a final fifth meeting in September 2021 to share our synthesis of the discussion at each workshop, feedback from the young people and our overall conclusions. Everyone invited to the initial four workshops was invited to join this meeting. Young people were invited to join this final meeting but preferred to comment on the initial findings in their own meetings with the research team, and for their views to be shared at the final workshop. This sense checking process resulted in some further refinements of the findings presented in this article. We are mindful that had different people attended the workshops or undertaken the synthesis of the findings that the conclusions might have been similar, but different. As such, what follows is a starting point for a discussion about what the future research agenda should look like.

## 4 FINDINGS

In our initial meetings with 15 young people with care experience to plan for the workshops, the key message was the importance of understanding the complexity of mental health and the recognition that mental health is not an isolated aspect of people's lives, or something that impacts a section of people's brains. It is deeply interwoven with individual's whole life experiences, of the past, the present and with anticipations, hopes and worries about the future. To truly support young people and understand their experiences, we need understandings of mental health that can capture this. Additionally, while young people talked about how we should think about mental health, key points were also made which related to how we should engage with young people and talk with them about mental health. In the meetings the young people repeatedly stressed that asking young people about their mental health needs to be founded on a sincere intention to listen to their experiences and to try and understand and support, seeing young people as partners in the process and involving them in how research knowledge is used to improve lives.

Fifty‐nine participants joined the four workshops (Table 1), some for more than one topic. The lowest represented group were policy makers, with overall a good mix of researchers and nonresearchers. This lack of participation by policy makers is important to note.

**TABLE 1:** Participants by country and role.

Participant country	Number	Participant role	Number
England	21	Academic/researcher	35
Ireland^a^	1	Advocate	13
Northern Ireland	5	Policy maker	2
Scotland	19	Practitioner	11
Wales	13		
Total	59	Total	61^b^

^a^ One participant from Ireland joined a focus group given their research was relevant to the focus of our work.

^b^ Some participants had more than one role.

**FIGURE 2:**
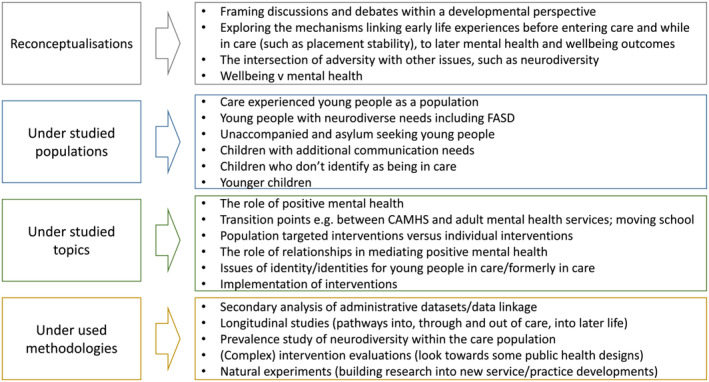
Core themes and research priorities.

Our synthesis of the discussions across the workshops and with the 15 young people identified four core themes (Figure 2). These themes represent dimensions of the future direction for research, and are interconnected. Any one study may address aspects of one or more dimensions highlighting the interconnectedness between theory, topics and research design.

### 4.1 Reconceptualizations

A key theme arising from the discussions related to how we conceptualized mental health. This is the bedrock upon which all other research must be built. While much of the existing research with people with care experience takes a broad definition of mental health, there were also concerns expressed that it was still rather limited, focusing primarily on concepts such as illness. Participants reported that there was a poorly considered approach to the more complex needs of neurodiverse young people who will also have experienced adversity and trauma. There was a need to consider the disruption of being placed in care and moving through the care system.

Our preliminary work with young people with care experience had highlighted the importance of thinking of mental health broadly. While young people were very cognizant of the importance of identifying and treating diagnosable neurodevelopmental and mental health disorders, they were keen to emphasize that mental health covered a wider spectrum of important aspects of their lives. Well‐being, of which positive mental health was one key element, was also important. In this regard friendships, relationships with caring and supportive adults, exercise, hobbies, rest and eating habits could all be considered as the building blocks for both compensating for and addressing the impact of some of the adversities experienced in life.

Workshop members also discussed that the experience of care is typically changing over time and that adopting a developmental approach to the study of the temporal aspect of well‐being was likely fruitful. This therefore lent itself to thinking about children's developmental trajectories and the variation that can occur between children for a variety of reasons. The importance of not over‐emphasizing a child's care status, or making blanket assumptions because of this status, were considered important, as this might detract from a more comprehensive assessment of their situation and needs. For example, not assuming that problematic or distressed behaviours are purely because of pre‐care trauma, or avoiding assumptions about attachment problems. There was agreement on the importance of thorough assessments, particularly as neurodivergence can be common in this group. At present, much of the existing research focuses on critical transition points such as entering care, changing placement and leaving care. These are all very important aspects of the care experience to know more about, alongside better understanding what these transition points mean for individual young people. However, it is very likely that the transition is mediated by the child's developmental stage and their developmental and mental health trajectory to that point and subsequently.

In the workshops, a recurring theme related to the need to develop models about the mechanisms for linking early life experiences before entering care and while in care, to later mental health and life outcomes. Such models could then be tested empirically, while also helping to think about how existing and new interventions could promote better future outcomes, and whether existing models of practice were fit for purpose. The increased interest in trauma‐informed approaches in working with individuals with care experience was considered potentially helpful, but of itself not wholly sufficient. Issues such as the experience of poverty, neglect, disability and neurodivergence rarely fit with existing trauma‐informed conceptualizations of children's needs. Also, there is a danger of terms being used routinely but in ways which are poorly defined and may undermine the importance of being able to use terms in a clinically relevant way to ensure appropriate interventions and treatments (Devaney et al., 2021).

### 4.2 Under‐studied populations

First, there was a consistent view that there is limited high‐quality research related to care‐experienced young people in general, particularly beyond describing the scale or nature of mental health problems experienced. Subsequently, there is a very slim evidence base related to understanding the mental health needs of particular subgroups of the care‐experienced population, how services and practitioners can support positive mental health, and how to respond effectively to mental health difficulties. There is a tendency to talk about the care‐experienced population as homogenous, with the main distinction being in relation to entry into care, time spent in care, the type of care provided and the process of exiting care (all of which are still important, but limited).

While further research on young people with care experience in general is needed, the workshops further identified subgroups within the care‐experienced population who might have particularly unique needs or require different types of intervention. Subgroups identified in the workshops were as follows: children who are unaccompanied and asylum seeking; children who are neurodivergent; younger (pre‐school) aged children; children adopted from care; and children subject to special guardianship. There was a consensus that we needed studies that looked at specific subgroups within the care‐experienced population (and how their needs might compare to children with similar characteristics not in care). Larger studies of the general care‐experienced population may also facilitate the exploration of the similarities and differences between subgroups.

This discussion raised an important point affecting research in this field in general. That is, the challenges of recruiting large and representative samples to answer more complex or fine‐grain questions. To do this requires strong partnership with organizations providing care for young people and collaboration between research teams. The need for data linkage and oversampling of subpopulations, such as those with neurodiversity, becomes critical. It would also be crucial that studies are appropriately funded given the need for some specific considerations, such as using translators.

Workshop attendees also discussed the clear need to better understand the interplay between neurodevelopmental conditions, mental health, and physical health for children generally, and children in care specifically. A large proportion of young people in care present with neurodevelopmental conditions, but this is often overlooked in mental health research on care‐experienced children, despite important implications for how such young people should be cared for, and helped. While not limited to children who are neurodivergent, but of particular importance to this group, there is the need for a much better understanding of cross‐cutting barriers and facilitators to these young people accessing existing services to address their mental health, including as they transition from child to adult services. This also includes how we might improve pathways between social care, education and mental health services, and between the statutory and the third sector.

There has, helpfully, been a significant body of research on the experiences of adolescents preparing to leave care and young adults post‐care experiences (Mann‐Feder & Goyette, 2019). This has been supplemented in recent years by work looking at therapeutic support for young people in residential care (Leipoldt et al., 2019). It was felt that this work could be enhanced by research looking at the mental health needs of young children entering care and in state care, most of whom, in the United Kingdom, are living with families, some of which are kin. For all the talk within children's social work of prevention and early intervention, there was little evidence of this translating into therapeutic support for children in a bid to stave off longer term psychological difficulties due to the adversities and traumas experienced. This would likely require the development of different, and possibly new, types of interventions for younger children, to those provided to adolescents through traditional child and adolescent mental health services and residential child care services. Participants also commented on the need for more integrated working between education, health and social services. Training offerings might be helpful, although the evidence to date of efficacy is mixed (Schoemaker et al., 2020).

Finally, there was a clear feeling among participants of the need to better understand the longer term outcomes for adopted children given the changing profile of which children are now adopted, and the concerns about the lack of ongoing support for a range of issues once an adoption order is granted.

The theme of under‐studied populations also connects back to the previous theme of reconceptualizations—in that we need to think about the characteristics, experiences and circumstances of every young person with care experience as being complex, dynamic and intersecting. Too many studies exclude young people because they are neurodivergent, they do not speak fluent English, or they are deemed too young. This is more a reflection of the scale of ambition and the often limited funding that is available for research. However, it was also felt to reflect a narrow perspective on what causes and sustains poor mental health, and who we should be most concerned about.

### 4.3 Under‐studied topics

The third core theme related to topics that appeared to be important but for which there was an insufficient amount of high‐quality research evidence to inform either understanding or practice.

A key message from the young people involved in our work was the need to focus on the factors promoting positive mental health and well‐being more generally. Many young people with care experience do benefit greatly from the care they receive from carers and mental health practitioners. However, there is a need to more fully understand what appears to work for some children, and whether this might provide insights into what could work for others. The young people highlighted the value they saw in thinking about how some young people are able to navigate the adversity they have experienced in life due to factors within themselves and also the wider context they are a part of. Better understanding the interplay between personal resilience and supportive environments appeared to be a fruitful area of future enquiry.

Another key issue identified by many people with care experience is the quality and continuity of relationships with others. Being able to understand how the quality of mental health was mediated by relationships with birth family, even if not living with them, with peers, with alternative carers and with professionals, was considered important in better understanding how relationships can both persist over time and also change, and the positive and negative consequences of such. Although the quality and continuity of relationships for young people is widely accepted as beneficial, we have less understanding of the essence of such healthy relationships and how they might be protected, strengthened or replicated for all young people with care experience, especially given the disruption caused to relationships by factors such as placement moves. Just as important was the need to understand how to support young people in managing negative, but persisting relationships, for example, with certain family members or acquaintances.

This leads to the issue of identity/identities for young people in care or formerly in care. Adolescence is a stage in life when the majority of young people question, cast and recast their identity. Workshop participants reported the added intricacy of having experienced care, with the stigma and complexity that this often involved. Supporting young people to explore and form their identity in relation to issues such as their ethnicity, sexual orientation and/or religious beliefs while being in the care of the state, and all of the additional scrutiny and limitations that this entails, would benefit greatly from research.

As mentioned previously, there is a need to better understand how to support young people in making key transitions in life, including, but not limited to, the experience of the admission to care, moves between placements, changes in schools and the transition out of care, which usually also involves transition between services for children and adults. Of course, some of these changes might not be predictable, but many can be anticipated and planned for. Therefore, what is the appropriate balance, for example, between preparing the individual with better coping strategies, and reimagining when and how transitions should take place, placing greater emphasis on the system rather than the individual. There are interesting examples of how services are seeking to make such transitions supportive, but less evidence of whether this has resulted in young people feeling better supported.

This acknowledgement led to discussion across workshops about the need to develop greater expertise in researching how interventions are implemented. Even where there was evidence for interventions, these were often not used in practice. Little is known about the conditions that typically moderate the effectiveness of similar interventions in different contexts or with heterogeneous populations, or what services need to be able to fully implement evidence‐informed intervention programmes. While it is the case that many mental health and social care services are chronically underfunded, the dearth of research investigating the impact of service structures on implementation of best practice, restricts advocacy efforts for capacity building. This is not just relevant to the delivery of interventions, but also in the use of routine outcome measures, and using existing standardized measures to allow for more thorough reflections on what works, with whom. Given our earlier comments, a better understanding of whether there are differentiated outcomes for particular subgroups within the care‐experienced population would be important.

Finally, the large numbers of children and young people in group living situations raised the question of what interventions could be developed that are aimed at groups rather than individuals and, relatedly, how they might draw on the expertise within multidisciplinary teams to be more cost effective, while also addressing young people's caution about engaging with individually focused interventions that might appear too threatening.

### 4.4 Underused methodologies and research designs

Our final set of findings relate to the types of methodologies and research designs that would usefully expand the range of questions and challenges that research could seek to address. Participants were able to articulate the benefits of particular research designs that they were most familiar with, but also to highlight the potential contribution that new or alternative designs could offer, even if that was not their area of expertise. It was acknowledged that undertaking research with young people with care experience is challenging. This is a result of the complex interplay between young people's own needs; their openness to engage in research given how busy and challenging life is already for them; the ability to follow up individuals no longer in state care, particularly those under the age of 18 years; and the desire by gatekeepers to minimize too many additional demands on young people, let alone the busyness of organizations. Although there are some positive examples of local authorities and other service providers agreeing to engage in new ways of working to evaluate their suitability and effectiveness in meeting the needs of young people with care experience, this was not a common occurrence. However, many service providers do routinely introduce changes to services and new ways of working and are often keen to get a sense of whether such changes are having the desired effect. One recommendation from the workshops was to seek to develop ways of building research into new service or practice developments, seeing these as natural experiments. Doing this from the outset was likely to generate better quality evidence rather than post‐hoc evaluations. However, it would require greater responsiveness by research organizations and funding for the research to be properly built into the business case and budget for the new service or intervention.

Second, while we have a good evidence base describing who enters care, their experience of the care system, and the journey beyond care, we have a more limited evidence base about the effectiveness of interventions, and how these benefit young people at a point in time and over time. There is a growing body of work in public health about developing and evaluating complex interventions that could usefully be considered (e.g., Skivington et al., 2021). This seeks to ask a broader set of questions than merely whether an intervention works or not (although this is still important to know), to focus on issues such as differentiating between an outcome and an impact, assessing the outcome relative to the resources required to achieve it, theorizing how the intervention works, and for whom in what circumstances, and how the intervention both is impacted by, but also contributes to system change. Approaches such as realist evaluations are methodologically robust and can be costly, but is likely to be more helpful for policy makers and service providers in the longer term. This could also lead to more robust approaches to economic evaluations, for example, building data collection for such approaches into the design of new services and interventions. It would though require researchers with the necessary skills for this type of research and analysis.

As we have stated elsewhere (Power et al., n.d.), such work would be helped by having a common approach to a core dataset that all studies would seek to capture to more fully allow for comparison and harmonization between studies. This is in line with current developments such as the ‘International Alliance of Mental Health Research Funders’. This consortium has emphasized the deleterious effects that the fragmentation of mental health research has had on the community, specifically pointing to the large number of measures that all use different methodologies—a point also noted by Flake and Fried (2020).

One of the glaring gaps identified within our workshops is the lack of a robust evidence base about the proportion of the care‐experienced population who are neurodivergent, and in what ways. Undertaking a comprehensive prevalence survey would be important for not only informing future research studies on a range of issues but also, surely, for policy development and service planning.

Internationally some countries have pioneered research approaches maximizing the linkage of data often routinely collected in the administration of public services. There is less of a history, and a much less well‐developed infrastructure in the United Kingdom around such data linkage (although this is improving especially in some UK nations). Recent attempts highlight both the potential, but also the limitations (Williams et al., 2020). Such approaches are likely to require further refinements in the infrastructure to support such data linkage across public services that often do not share data in these ways (e.g., health, housing, criminal justice and education), and the development of a cadre of social science researchers with the requisite skills to undertake the sophisticated analysis that would then be possible.

Finally, the United Kingdom has a long history of birth cohort studies, and these have been supplemented in more recent years by some longitudinal studies of children in care (e.g., Biehal et al., 2020; McSherry et al., 2010). We now have a clearer sense of the benefit of both approaches. For example, population wide cohort studies allow us to compare the trajectory of all children over time and to look for variations in subgroups. However, within these studies, the numbers of care‐experienced young people are relatively small, with attrition over time, even if the survey has sought to collect regular information about care status at each sweep. This limits the ability to truly explore outcomes and to meaningfully link datasets. Oversampling and having an agreed core set of questions to be asked of those in care or previously in care would aid greater understanding of the pathways and trajectories of care‐experienced individuals against their noncare‐experienced peers, while also facilitating sub‐analysis of specific groups of care‐experienced individuals and more similar groups of noncare‐experienced individuals.

Ultimately, producing more research without the requisite mechanisms and support for services and policy makers to adopt the research undermines the ability to ensure that research can enhance the future development of effective supports for young people.

## 5 DISCUSSION

Invariably, in seeking to identify a future research agenda for a whole area of practice, one is struck by the scale of the ambition and how limited this first foray is. Our intention is not to constrain research efforts but rather to see greater focus on those ideas, issues and approaches that might lend greater support to policy makers, service providers and practitioners in supporting the mental health of young people with care experience. We are mindful though of the lack of a greater number of policy makers in our workshops, and we hope to take forward individual meetings in the time ahead.

What was apparent from the workshops, and across participants, was the need for a stronger focus on understanding and appropriately responding to the mental health of young people. In this respect research is an important aid. However, as Bakketeig et al. (2020) state, it is important to start with the outcomes that young people deem as important. Our work highlights the value and importance of being alongside young people with care experience as active participants in the research process and adopting methods of involvement that work primarily for them.

Many current definitions and interpretations of mental health use medical concepts and language and tend to problematize the individual and take an almost entirely negative view of mental health. However, the young people informing our work defined mental health more closely to that stated by the World Health Organization (2022) as a state of mental well‐being that enables people to cope with the stresses of life, realize their abilities, learn well and work well and contribute to their community. Mental health is a basic human right. And it is crucial to personal, community and socio‐economic development. This encompasses neurodiversity and mental health disorders, while also resisting the temptation to see mental health through the lens solely of disorders. A balance must be struck between recognizing that a high proportion of care‐experienced individuals will experience mental disorders, while also seeking to adopt more preventative and early intervention approaches that reduce individuals experiencing severe mental illness, while also being responsive to those experiencing such poor mental health.

A key finding arising from our work is the need to move beyond seeing the care‐experienced population as a single entity, differentiated mainly by age, circumstances leading to care and the type of care provided. To more fully engage with the heterogeneity of the care population, the complexity of their experiences before, during and after their time in care, and the intersection of their various characteristics in mediating many of these issues, will require greater thoughtfulness and ingenuity in how research studies are designed and undertaken. This in itself holds the potential to better reveal the factors at play at individual and systemic levels regarding the factors promoting or impacting on the mental health and well‐being of individuals.

More robust research will require investment in terms of increased funding to undertake the work, as well as in ensuring that there is a suitably skilled cadre of researchers able to use the more sophisticated techniques proposed. One of the benefits of our own approach was the interdisciplinary nature of the research team, with expertise across topics, methods and disciplines. This opened the conversations within the project and at the workshops to a wider range of views and contributions that has been helpful. Mental health is important to us all, and there are contributions to be made from a diverse set of perspectives and vantage points.

A key finding is that the existing large body of high‐quality and robust qualitative research on the mental health of young people with care experience could be supplemented by using a range of other research designs and methodologies. This would improve our understanding of how individual's mental health changes over time, while also helping us to better understand the factors that might contribute to recovery from adversity and trauma. This could be through individual therapies, improvements in the contextual support for young people in schools and placements and improvements regarding how all young people are supported regarding their mental health and well‐being. Using more quantitative or experimental methods has the potential to help us move beyond describing what is happening in young people's lives, to more accurately deciding how to intervene, and which interventions are likely most helpful for specific individuals and groups.

## 6 CONCLUSION

Positive mental health is a complex interaction between the individual and their environment, even in situations where there may be a genetic component to an individual's susceptibility to develop a mental health condition. We therefore have a responsibility as a society to create the conditions that promote positive mental health, especially for those for whom life events and circumstances are more heavily stacked against them. The recent independent reviews of children's social care in England (McAllister, 2022) and Scotland (Duncan, 2020) support such an approach.

We see this article as the continuation of a conversation begun in the workshops in 2021 and hope that others will accept the invitation to discuss the four themes we have identified, and the specific foci proposed by participants. We hope that those who commission and fund research will also further engage in these discussions. There is a growing realization that improved mental health for everyone, including those with care experience, will often lead to improvements of other important indicators of a well‐functioning society such as engagement in education, training and employment, lower rates of anti‐social and criminal behaviour, decreased levels of poor physical health, and most importantly, improved quality of life.

## Data Availability

Author elects to not share data.
